# Beaver exploitation, 400,000 years ago, testifies to prey choice diversity of Middle Pleistocene hominins

**DOI:** 10.1038/s41598-023-46956-6

**Published:** 2023-11-13

**Authors:** Sabine Gaudzinski-Windheuser, Lutz Kindler, Wil Roebroeks

**Affiliations:** 1 MONREPOS Archaeological Research Centre and Museum for Human Behavioural Evolution (LEIZA), Schloss Monrepos, 56567 Neuwied, Germany; 2 Institute of Ancient Studies, Pre- and Protohistoric Archaeology, Johannes Gutenberg-University Mainz, Schönborner Hof, Schillerstraße 11, 55116 Mainz, Germany; 3 Faculty of Archaeology, Leiden University, P.O. Box 9514, 2300 RA Leiden, The Netherlands

**Keywords:** Evolution, Archaeology

## Abstract

Data regarding the subsistence base of early hominins are heavily biased in favor of the animal component of their diets, in particular the remains of large mammals, which are generally much better preserved at archaeological sites than the bones of smaller animals, let alone the remains of plant food. Exploitation of smaller game is very rarely documented before the latest phases of the Pleistocene, which is often taken to imply narrow diets of archaic *Homo* and interpreted as a striking economic difference between Late Pleistocene and earlier hominins. We present new data that contradict this view of Middle Pleistocene Lower Palaeolithic hominins: cut mark evidence demonstrating systematic exploitation of beavers, identified in the large faunal assemblage from the c. 400,000 years old hominin site Bilzingsleben, in central Germany. In combination with a prime-age dominated mortality profile, this cut mark record shows that the rich beaver assemblage resulted from repetitive human hunting activities, with a focus on young adult individuals. The Bilzingsleben beaver exploitation evidence demonstrates a greater diversity of prey choice by Middle Pleistocene hominins than commonly acknowledged, and a much deeper history of broad-spectrum subsistence than commonly assumed, already visible in prey choices 400,000 years ago.

## Introduction

A solid understanding of early hominin diets, key for tracking human behavioral and cognitive evolution, is hampered by the fact that the archaeological record is strongly biased towards the remains of large ungulates, while it is well-established that a reliance on (relative lean) game meat alone would not have provided a sufficient subsistence base given human dietary needs^1,2^. Despite taphonomic and site-preservation biases^3^, recently various studies have documented a greater diversity in hominin food choices, including regular exploitation of a variety of small animals, plant and aquatic (freshwater and marine) foods, not only for the early modern human lineage in Africa^4–6^, but also for Neanderthals, be it mainly from the southern parts of their range^3,7–10^. Most of that evidence dates respectively to the Middle Stone Age of Africa and to the later Middle Palaeolithic in Europe, from about 125,000 years ago (125 ka) onwards. Far less is still known about the subsistence base of the Middle Pleistocene predecessors of both lineages (e.g.^10–12^), with that record still strongly suggestive of a narrow, large- and medium-sized ungulates focused subsistence base^13^.

**Figure 1:**
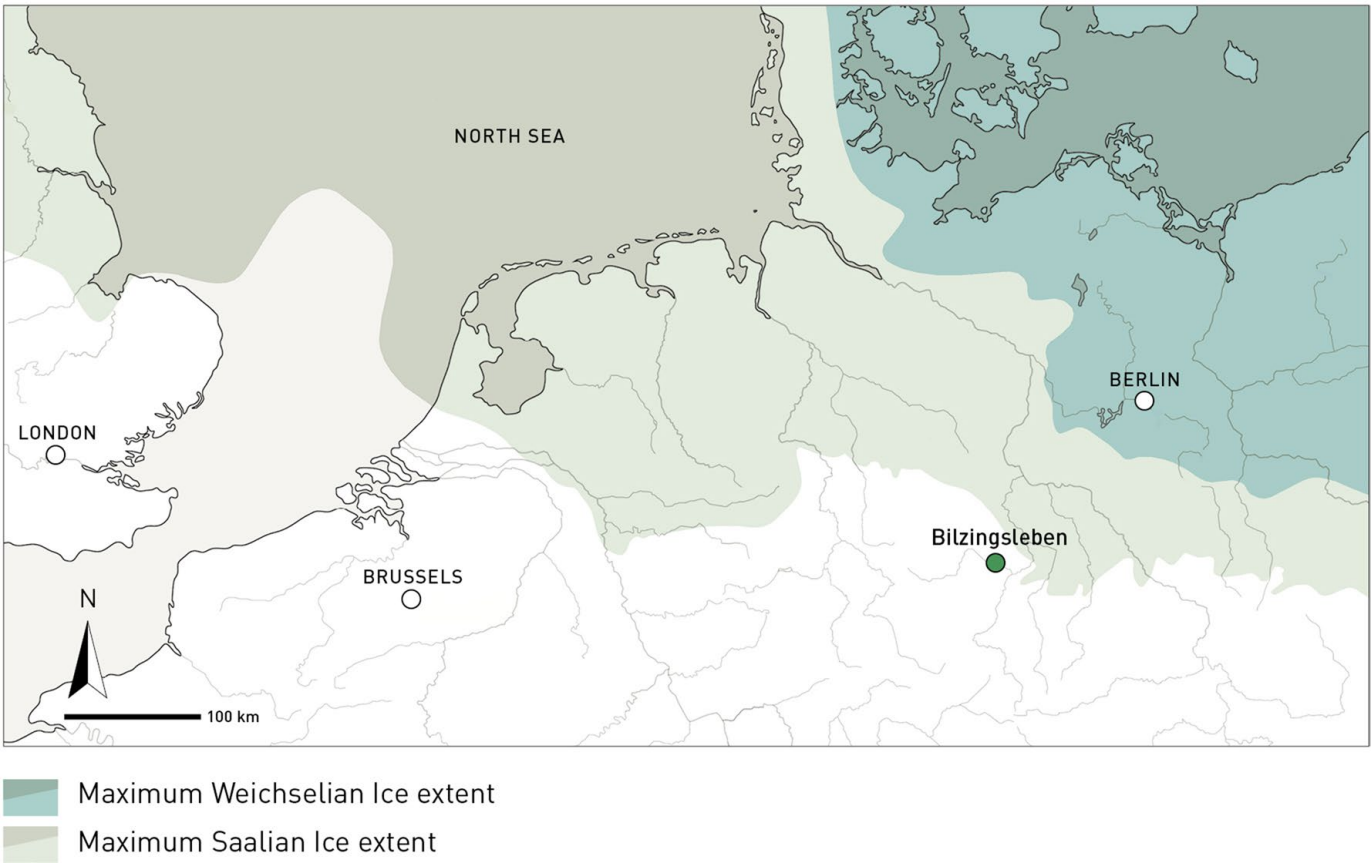
Location of Bilzingsleben on the northern European plain, relative to the maximum extent of the Saalian and Weichselian glaciers. Both ice-sheets ended north of the Bilzingsleben site, enabling preservation of the locale.

Here we present new data of particular relevance for our knowledge of Middle Pleistocene diet breadth, from the ca 400 ka old (Marine Isotope Stage [MIS] 11) hominin open air site of Bilzingsleben, in central Germany (Fig. 1) (Supplementary Information Text 1), with, as shown below, abundant evidence for systematic exploitation of beavers. Our study was triggered by a recent analysis of the rich Bilzingsleben beaver assemblage by Heinrich and Maul^14^, who established a prime-age mortality profile for the *Castor fiber* remains from the site, leading them to infer that these individuals had been targeted by human hunters. The beaver material from Bilzingsleben mainly derives from *Castor fiber*, the Eurasian beaver, a close relative of *Castor canadensis,* a key prey species of North American hunter-gatherers, much sought after for its fat and fur. *Trogontherium cuvieri* is also present in the Bilzingsleben material, be it in much smaller numbers (see below and Table 1). This extinct beaver was of similar size as the extant Eurasian beaver^15^, and often found in the same Pleistocene deposits, though it is unclear whether they shared the same habitat or occupied slightly different ones^14^. *Castor fiber* is currently the largest rodent in Europe, with very strict ecological preferences, which include the presence of sufficiently large water bodies and a dense tree cover. This keystone species influences the local hydrology and its environment. For the British record it has been suggested that early Holocene foragers were attracted to beaver territories with their well-maintained water bodies surrounded by abundant wood resources^16^.

**Table 1:** Species representation in the Bilzingsleben beaver material.

Specimen	*C. fiber*	*T. cuvieri*	Beaver	Total
Bone	311 (64%)	5 (1%)	172 (35%)	488 (100%)
Mandible/maxilla with teeth	42 (93%)		3 (7%)	45 (100%)
Isolated incisor	148 (36%)	221 (54%)	38 (9%)	407 (100%)
Isolated molar	1257 (89%)	139 (10%)	24 (2%)	1420 (100%)
**Total**	1758 (74%)	365 (15%)	237 (10%)	2360 (100%)

Next to a rich Holocene record of beaver exploitation in Eurasia and northern America, there is some scattered evidence for exploitation of beaver in the later phases of the Middle Palaeolithic. It mostly dates from the relatively short Last Interglacial (around 125,000 years ago), when a warm temperate climate and forested conditions provided ideal habitats for these semi-aquatic animals in major parts of Eurasia^17^. For earlier warm-temperate phases, data were virtually absent thus far, with no convincing evidence for regular exploitation of these animals, despite being of such great importance for recent hunter-gatherers in the northern parts of Eurasia and North America. (Supplementary Information Text 2).

## Results

We analyzed 2,496 remains of *Castor fiber* and *Trogontherium cuvieri,* recovered during Dietrich Mania’s long-term (1969–2002) excavations at Bilzingsleben, covering an area of approximately 1800 m^2^ (Supplementary Text 1). *Castor* and *Trogontherium* were of similar size and display different limb bone proportions, but only the skull morphology and teeth (molars and incisors) allow for clear distinction of both species^15,18^. Out of 1146 molars, Heinrich^19^ identified 1016 (89%) as belonging to *Castor* and 130 (11%) as *Trogontherium*. From these molars he calculated a Minimum Number of Individuals (MNI) of 82 for *Castor* and a MNI of 12 for *Trogontherium*. Species attribution is unevenly distributed with respect to different tooth types and bones (Table 1). In contrast to the molars, more than half of the incisors were identified as *Trongontherium.* The fragmentary state of the long*,* rootless beaver incisors in the Bilzingsleben material inhibits any estimate of Minimum Numbers of Elements (MNE) and individuals (MNI). Almost all mandibular and maxilla fragments containing teeth are attributed to *Castor fiber,* as well as about two-third of all bones and bone fragments. However, only 5 out of a total of 488 bones could unambiguously be assigned to *Trogontherium,* leaving about one third of all remains unidentifiable at the species level. As our study focused on anthropogenic marks on beaver bones, we lumped all bones together under the taxon “beaver” in the skeletal representation, acknowledging that most of the specimens belong to *Castor fiber*.

With 2496 specimens (1963 teeth and 533 cranial and postcranial bones and bone fragments) the Bilzingsleben material represents—to our knowledge—by far the largest beaver assemblage of the Pleistocene.

While the MNI calculated based on teeth is 94, the post-cranial MNI is considerably lower, with 36 for the tibia (Table 2). Only the mandibles and long bones occur in high numbers in the Bilzingsleben material. The paucity of bones from the thorax, the zonoskeleton and to a lesser degree from the autopodium may be related to a recovery bias against smaller elements and/or post-depositional destruction. Body parts with a high nutritional value are better preserved than elements with a lower meat yield (see^20^). Against this, an unbalanced representation of the long bones, with 72% coming from the hind leg and only 28% from the front leg is noteworthy, given that the thigh comprises one third of carcass weight. Unfortunately, data on bone density is limited^21,22^, inhibiting further analyses and identification of the various taphonomic processes and actors involved in the formation of the beaver assemblage.

**Table 2:** Skeletal element representation for the studied sample.

	Specimen	NISP	MNE	MNI	MNEcut
Head	Cranium/Maxilla	3	3	2	
	Mandible	57	45	26	6
	I sup/inf	407			
	I inf	16			
	P/M sup	119	119		
	P/M sup/inf	1145	1145	77–94	
	P/M inf	276	276		
Torso, Tail, Zonoskeleton	Vert cerv	1	1	1	
	Vert tho/lumb	3	3	1	1
	Vert lumb	5	5	2	1
	Vert lumb/caud	1	1		
	Vert caud	9	9	1	
	Rib	18	17	1	
	Scapula	14	10	6	1
	Pelvis	20	17	10	2
Stylo-, Zeugopodium	Humerus	37	21	13	5
	Radius	10	7	5	1
	Ulna	22	17	11	3
	Femur	70	43	23	13
	Tibia	108	67	36	6
	Fibula	1	1	1	
Autopodium	Carpals	4	4	1	
	Carpals/Tarsals	1	1	1	
	Astragalus	28	28	17	
	Calcaneus	19	18	12	4
	other Tarsals	7	7	2	
	Metapodial	42	29	5	3
	Phalange 1	9	9	1	
	Phalange 2	5	5	1	
	Phalange 3	5	5	1	
Other	indet	34			
	Total	2496	1913	77–94	46

NISP—Number of Identified Specimens, MNE—Minimum Number of Elements, MNI—Minimum Number of Individuals, MNEcut -Minimum Number of cut marked Elements.

### Anthropogenic modifications

**Figure 2:**
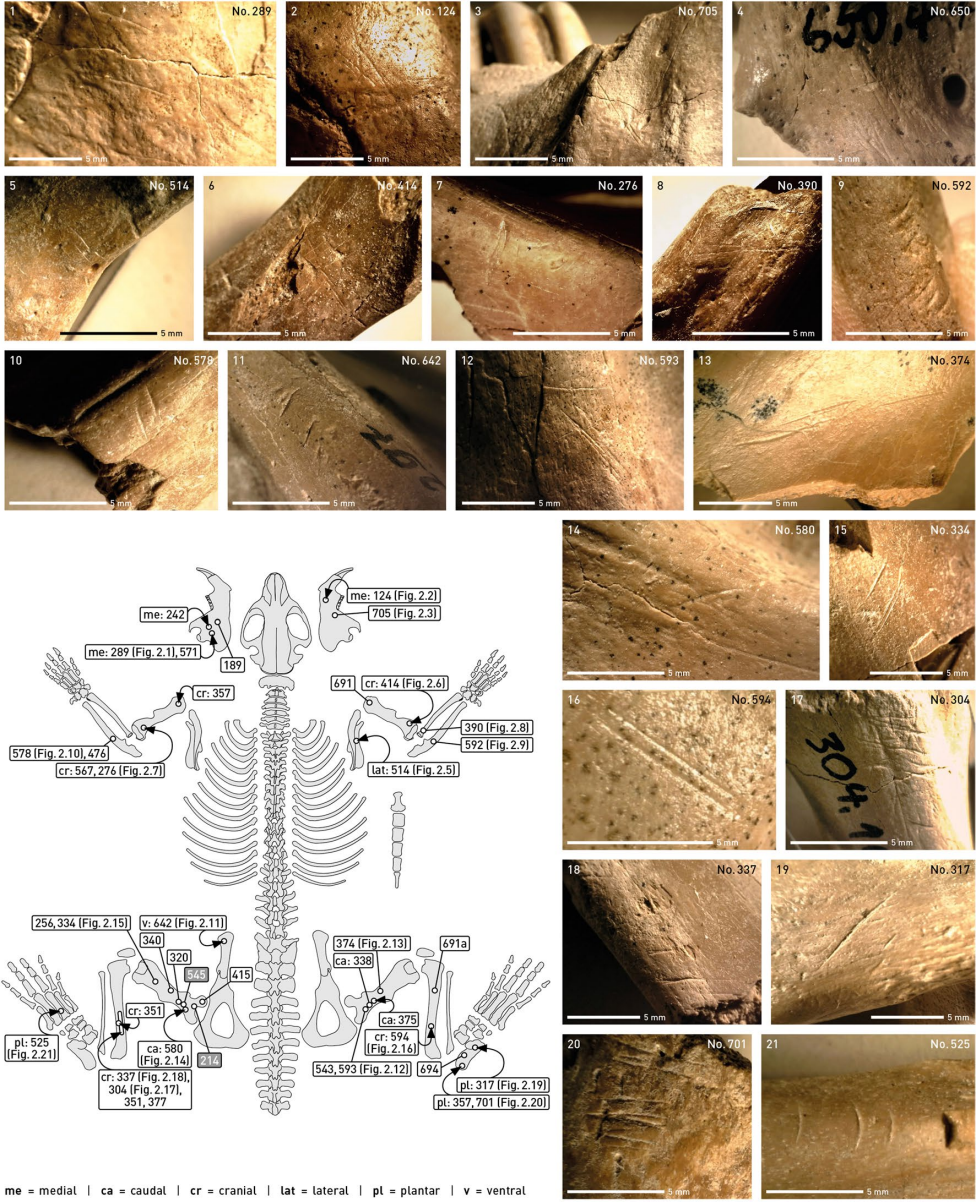
Cut marks on *Castor fiber* skeletons (illustrations are arranged according to body parts) and their anatomical position. The numbers denote the inventory designations of individual bones with cut marks. For a detailed description see text.

Excellent bone preservation of the beaver remains enabled the documentation of cut marks which were identified on various body parts (see Table 2 and Supplementary Table 1). They attest to the exploitation of complete carcasses, most traces documenting skinning and subsequent disarticulation. Cut marks indicative of meat exploitation could only be observed with any certainty on the femur (Fig. 2).

Traces of skinning can be clearly observed on the mandibles. On right and left specimens cut marks occur on the lateral face of the bones (e.g., Find Number [No] 289, Fig. 2.1) as well as medial basal (e.g., No 124, Fig. 2.2). Numerous cut marks can also be observed on the distal diaphysis of the tibia (e.g., No 594, Fig. 2.16; No 304, Fig. 2.17; No 337, Fig. 2.18), interpreted as traces of skinning in beaver^23^. Accordingly, cut marks on the articulating calcanei, located dorsally, above the posterior talar facet of the bone (No 694), dorsal proximal (e.g., No 701, Fig. 2.20; No 357), plantar proximal (e.g., No 317, Fig. 2.19; No 741) and plantar medial-distal (No 317) can be related to this step of carcass exploitation. Finally, deeply incised cut marks on the plantar mid-shaft of an mtIII (No. 525, Fig. 2.21) can also be clearly associated with beaver skinning.

The separation of mandibles from the crania is attested by cut marks on the medial face of the mandibles, in the area of the foramen (e.g., No 705, Fig. 2.3). Vertebrae are strongly underrepresented in the present sample, but even here, cut marks on a vertebral body document the breaking up of the beaver carcasses (e.g., No 650, Fig. 2.4), as do cuts on the lateral collum scapulae (e.g., No 514, Fig. 2.5). A cut mark ventrally on the os ilium of a pelvis (No 642, Fig. 2.11) can also be interpreted in this manner. Cut marks on proximal and distal humeri (e.g., No 414, Fig. 2.6; No 276, Fig. 2.7) and on a proximal radius (No 390, Fig. 2.8) attest to the disarticulation of the forelimb, as do cut marks lateral on the proximal ulna, in the area below the articulating joint with the humerus (e.g., No 592, Fig. 2.9; No 578, Fig. 2.10). Deboning of the hind leg is indicated by cut marks on the proximal epiphysis of a left femur (No 415).

The upper part of the femur is the only part of the entire carcass with traces clearly associated with meat removal. These cut marks are located cranially, only on the upper/middle part of the diaphysis (e.g., No 642, No 593, Fig. 2.12; No 374, Fig. 2.13; No 580, Fig. 2.14; No 334, Fig. 2.15). Cut marks were also found on the small number of bones identified as *Trogontherium*: cut marks plantar, on the proximal diaphysis of a left mtIII (Sq 391, no findnumber) document the skinning of the animals, while cut marks on the cranial surface of a right proximal femur (No 214) testify to the disarticulation of the hind leg. A left femur (No 545) has cut marks in the proximal/medial area of the cranial diaphysis, testifying to the defleshing of the bone. The overall distribution of cut marks indicates that *Trogontherium cuvieri* was exploited in the same way as *Castor fiber.*

## Discussion

The cut mark data presented here provide additional strong and independent evidence in support of an anthropogenic origin for the Bilzingsleben beaver assemblage hypothesized by Heinrich and Maul on basis of their age profile data^14^, and importantly demonstrate repeated hunting and exploitation of beavers around 400 ka. On basis of the molar teeth, Heinrich^19^ calculated an MNI of 82 for *Castor* and 12 for *Trogontherium* (see also^14^); our bone (tibia) MNI is 36 and cut marked bones yield an MNI of 8 (Table 2**, **Supplementary Table 1).

Based on the cut mark distribution pattern, beaver may have been targeted for their skins, as well as for their meat. The paucity of cut marks unambiguously related to meat removal does not necessarily indicate a neglect of beaver food resources. In Holocene beaver assemblages the frequency of fileting marks is much lower than cut marks indicative of skinning and dismemberment^23–25^. Muscle tissue around the bones could probably be gnawed away easily instead of being sliced with stone tools, especially when the tissue is softened, e.g., via cooking or fermentation. However, apart from some diaphyseal cylinders and an underrepresentation of epiphyses, direct evidence for hominin gnawing on bones, such as toothmarks observed on rabbit bones^26–28^ , is missing.

In historical times, traditional hunters highly prized beaver meat and fat: about 50% of the weight of the animal, i.e. 10 kilos of an average beaver of 20 kilos, was consumable^25^ (meat, fat, innards). The meat was rich in proteins, iron and minerals, while its fat contained 48.8–53% of PUFAs^29^. The beaver’s tail is remarkably large in relation to body size, and plays an important part in the storage of energy reserves in the form of fat—a key resource for foragers—the content of which changes strongly with the seasons^29,30^, with the volume doubling through grease storage between spring and autumn^31^. Beavers also provided castoreum, produced by their glands, a substance employed as a universal medicine at least since Antiquity, and used by traditional hunters as a lure to attract a range of prey animals^32^. Regular beaver exploitation and consumption at Bilzingsleben thus made nutritional sense, as: it added valuable macro- and micro-nutrients to the diet of Middle Pleistocene hominins.

Beavers are monogamous and territorial animals, with home ranges of between 1 and 3 km along water streams. The beaver lodge is generally inhabited by the parents and two generations of offspring. With sexual maturity the young adults are expelled and start to create their own territory and lodge. The mortality analyses for *Castor* provided by Heinrich and Maul^14^, show that most individuals died in this crucial stage of young adulthood, when individuals become driven from the lodge, roam longer on land in search of their own territory and are inexperienced and thus more vulnerable to predation than  older adults. The dominance of this age class in the Bilzingsleben assemblage can be interpreted as the result of repetitive hominin predation on young adult beavers over an extended period. With their semi-aquatic lifestyle, beavers call for hunting tactics that are different from those for larger terrestrial animals. While ethnographic and historical sources show that beavers were mostly caught by means of nets or traps, often installed at the exits of their lodges^25^, the focus on young adults documented at Bilzingsleben suggests more specific individual-targeting hunting practices^14^.

The wider Bilzingsleben faunal assemblage suggests an abundant availability of large game animals, with age profile data, skeletal element representation, and evidence regarding modification of bones (cut marks), among other evidence, demonstrating the exploitation of rhinoceros and bears^33^. The sheer numbers of medium and large mammal fossils accompanying the beaver remains do not suggest that resource depletion of large mammal game forced hominins to shift to smaller animals. The archaeozoological evidence does not allow us to infer specific reasons for repetitive beaver harvesting at this locale, i.e. whether these were nutritional (protein and fat focused) in character, whether they went for the skins and/or the castoreum, or a combination of these.

### Beyond Bilzingsleben

The Bilzingsleben beaver data nuance our large-ungulates biased picture of Lower Palaeolithic subsistence activities and increases our knowledge of the dietary breadth of Middle Pleistocene, Lower Palaeolithic hominins in the northern part of their range in temperate Europe. The data not only constitute the earliest evidence for systematic smaller game (large rodent) exploitation north of the European mountain chains, but also the earliest unambiguous case of Pleistocene beaver exploitation thus far. The large beaver assemblage of Bilzingsleben and its cut mark record suggest full carcass exploitation as well as prey selection over a prolonged period, as demonstrated by the mortality profile. It needs to be emphasized that these data could only be obtained through Dietrich Mania’s long-term large-scale excavations during more than 40 years of field work, which enabled the recovery of such a quantity of material. Besides a good state of bone preservation, a large sample size is crucial for identifying hominin subsistence patterns in zooarchaeological assemblages.

Furthermore, hominin prey choices are probably reflected differently in rock shelter sites—to which prey or parts thereof were often transported—versus open air settings such as at Bilzingsleben, where primary butchering and processing tended to be more important, and hence better visible than in rock shelter settings. Presumed differences in prey choices between southern and northern parts of Europe may not so much result from hominin behavior per se as from such large-scale site formation differences—with karstic settings dominating in southern Europe.

The large numbers of animals and cut marked specimens indicate that beaver exploitation at Bilzingsleben was a common part of subsistence habits and diet at 400 ka in this area. At the same time, our study also provides a new context to revisit and interpret the thus far very anecdotal *Castor* evidence from roughly contemporary sites, such as the few cut marked bones from Caune de l’Arago in southern France^17^**,** the unmodified but transported material from Gruta Aroeira and Gruta da Oliveira, both in Portugal^34^, and the scarce beaver remains from earlier sites in the Atapuerca, Spain, site complex^35^. Their presence in Iberia during the Middle Pleistocene, and their occurrence in the Caune de l’Arago sequence suggest that far from being an exclusively “northern” rodent, exemplified by the Bilzingsleben material, beaver populations may have been well-established in the western part of the Mediterranean in the Middle Pleistocene.

Unlike small mammals such as leporids, recently shown to have played some role in hominin diets in the northwestern Mediterranean from 400 ka onward^13^, beavers were high-ranked prey animals, a valuable resource for (sub-recent) hunter-gatherers in Eurasia and North America from the early Holocene onward, clearly demonstrated through their abundant remains in archaeological assemblages. The Bilzingsleben data suggest a similar importance during earlier warm-temperate periods in Europe, from at least 400 ka onwards, and more generally, indicate that “Broad spectrum diets” may have been present much earlier than generally assumed^13^.

## Methods

### Taxonomic determination

Based on previous research by Fischer^36^, Günther^37^ and Heinrich^19,38,39^ the material stored in the Landesamt für Denkmalpflege und Archäologie Sachsen-Anhalt (Halle, Germany) is sorted by bone type and species name, *Castor fiber*, *Trogontherium cuvieri* or just beaver without species attribution. From the material retrieved from the Pasda excavations stored in “Bilzingsleben Sammlung” of the Friedrich Schiller University at Jena (Germany), we were able to identify 110 beaver bone and teeth specimens, nondiagnostic for any further species attribution.

### Number of identified specimens per taxon (NISP), minimum number of elements (MNE) and minimum number of individuals (MNI)

All beaver remains in the Halle material were already sorted by skeletal element previously, while we identified the specimens in the Jena collection that had not yet been diagnosed by Müller^40^. Every fragment was counted individually; in the case of mandibular and maxilla fragments containing teeth, every tooth was counted separately, in order to create comparability to the high number of isolated molars in the analysis. This procedure resulted in a total of 2496 beaver specimens (NISP) in the Bilzingsleben material. The MNE was derived from recording and counting the location of a fragment in the respective complete bone, e.g., the long bones were subdivided in proximal and distal epiphyses, upper, middle and lower shafts with anterior, posterior, lateral and medial faces. In addition the state of epiphysial fusion was recorded (joint fusion occurs in most beaver bones throughout adulthood and is not indicative of a juvenile age class^41^). The MNI calculated from postcranial material was derived from the MNE considering the specimen's side and state of fusion.

Beaver dentition consists of a deciduous premolar, which is replaced by a P4 and three molars, M1–M3. Only a minor portion of the molars can be accurately assigned to their appropriate location and side in the jaw. Out of 1520 cheek teeth 1142 could not be attributed to represent a certain molar, while the remaining 278 teeth are attributed to type and/or side and/or to the upper or lower toothrow. In sum the Bilzingsleben molars can belong to 5 different teeth, in 4 different locations (upper and lower jaw, left and right side). Lumping all molars together and assuming an even distribution of all molar types an MNI of 77 can be calculated. Also assuming an even distribution of all unidentified molars but in consideration of the identified molars the MNI rises up to 94, which is in accordance with the MNI calculated by Heinrich^19^.

### Bone surface modifications

Bone surface modifications were studied using hand-held lenses with a magnification of up to 10x, a Dino-Lite PRO digital microscope with a magnification up to 200 × and a Leica reflected-light microscope with a magnification of up to 32x. For each bone or bone fragment, the location of the observed traces on the bone were photographed with a NIKON camera, while close-ups of the bone surface modifications were produced with the Dino-Lite PRO equipment. Traces caused by biotic and abiotic agents were identified using the taphonomic collection of the Archaeological Research Centre and Museum for Human Behavioural Evolution, MONREPOS, as well as diagnostic criteria published by Fernández-Jalvo and Andrews^42^.

### Supplementary Information

Supplementary Information.

## Data Availability

The beaver materials used in this study are held by the Landesamt für für Denkmalpflege und Archäologie Sachsen-Anhalt (Halle, Germany) and the “Bilzingsleben Sammlung” of the Friedrich Schiller University at Jena (Germany). All data are presented in the main text and the Supplementary Information.
