# Supplementary material for: Beaver exploitation, 400,000 years ago, testifies to prey choice diversity of Middle Pleistocene hominins
Source: Sci Rep. 2023 Nov 13;13:19766. doi: 10.1038/s41598-023-46956-6 (PMC10643649; doi:10.1038/s41598-023-46956-6)
Supplement: Supplementary file 1 — Supplementary Information. [file 41598_2023_46956_MOESM1_ESM.pdf]

Beaver exploitation, 400,000 years ago, testifies to prey choice diversity of Middle Pleistocene hominins

Sabine Gaudzinski-Windheuser [1,2], Lutz Kindler [1,2], Wil Roebroeks[3*]

[1] MONREPOS Archaeological Research Centre and Museum for Human Behavioural Evolution (LEIZA), Schloss Monrepos, 56567 Neuwied, Germany

[2] Institute of Ancient Studies, Pre- and Protohistoric Archaeology, Johannes Gutenberg-University Mainz, Schönborner Hof, Schillerstraße 11, 55116 Mainz, Germany

[3] Faculty of Archaeology, Leiden University, P.O. Box 9514, 2300 RA Leiden, The Netherlands

*Corresponding author: w.roebroeks@arch.leidenuniv.nl

SUPPLEMENTARY TEXT 1: THE BILZINGSLEBEN SITE

The Bilzingsleben archaeological site is situated in the Wipper Valley, a tributary of the Saale river in central Germany. The site was embedded in sand and lake carbonates of a travertine spring, which subsequently covered the archaeology yielding deposits with a 3-4 m thick sequence of solid travertines, exploited for building material. Extensive excavations took place between 1969 and 2002 by Dietrich Mania, over a total area of about 1800 m^2; small-scale excavations by Clemens Pasda followed from 2004 to 2007 [1-4]. More than three decades of excavations yielded large numbers (> 100,000) of stone artifacts, tons of vertebrate bones and bone fragments, famously including hominin remains (NISP 28, MNI 3) assigned to *H. erectus bilzingslebensis* [5], the fragmentary nature of which makes them difficult to assess[6]. The vertebrate fauna comprises 54 species, including rhinos, elephant, bear, wolf, cervids, bovids, horse, primates (besides *Homo*: *Macaca florentina*), a wide range of small mammals as well as *Castor fiber* and *Trogontherium cuvieri.* The site also yielded large amounts of molluscs, wood fragments, pollen and a wide range of leaf imprints. Floral remains and the rich fauna are indicative of a warm-temperate climate, which on the basis of biostratigraphy and some chronometric (U-series and ESR) data is younger than the Cromerian and Elsterian complexes but older than the Saalian glaciation, and correlative to Marine Isotope Stage 11, with an estimated age of 374 to 424 ka (see e.g. [3,7] for further discussion of the age range estimates for the site).

In Mania's interpretation of the Bilzingsleben evidence[2], the archaeological finds come from a small peninsula-like shore terrace edge (*Uferplatte*), of an approximately 200 by 300 m large lake, with primary context traces of hominin occupation). This edge is bordered by an alluvial fan close to the travertine spring, made up of friable travertine sands (*Schwemmfächer*) that contained material reworked from occupations on the *Uferplatte.* The finds come from both the shore and the alluvial fan facies. There is debate on the specifics of the site formation processes and the interpretation of the spatial patterns at the *Uferplatte*: according to Mania and his team the archaeological material there is preserved in primary context, including *in situ* remains of a number of dwelling structures, whereas Pasda and colleagues have suggested that the site contains reworked and redeposited cultural and natural materials formerly scattered in the surrounding landscape (see discussion in [3,8]). However, there is little doubt

about the homogeneity of the rich fauna in terms of it belonging to one warm-temperate phase.

A recent study by Brasser [4] focused on the megaherbivores from the site as well as its bear remains, investigating a possible anthropogenic impact through the analysis of > 8200 skeletal remains, of minimally 245 individual animals (*P. antiquus* MNI = 23, *Stephanorhinus kirchbergensis/hemitoechus* MNI = 112, *B. priscus/B. primigenius* MNI = 22, *Ursus* sp. MNI = 88) which highlighted the complicated taphonomic history of the Bilzingsleben fauna. According to Brasser, the faunal assemblage formed over a relatively short period and contains a mixture of animals that died of natural processes, including carnivore kills, and as a result of human hunting, with the bones of bears and rhinos showing traces of human interference in the form of cut marks. The general paucity of cut marks identified in Brasser's study may be due to abrasion which altered bone surfaces and erased cut marks on bones from larger taxa faster than on bones from smaller taxa, as suggested by taphonomic experiments [9,10] and underlined by the good cut mark evidence from the beaver assemblage presented in this study.

Spatial distribution of beaver remains

The excavations at Bilzingsleben were carried out using a 1.5 by 1.5 m grid system (*Planquadrate*) and extended over an area of approximately 1,800m^2. As mentioned above, Mania separated two distinct depositional areas, in the northern part – the alluvial fan (*Schwemmfächer)* and the terrace edge (*Uferplatte)*. All archaeological finds, including the beaver bones, are labeled according to their square of origin and find number. In a previous study, Heinrich [11] reported two main areas of deposition for beaver teeth, at the transition from the alluvial fan to the terrace edge, and in the area of the "working zones" identified by Mania in the terrace edge area. In Heinrichs interpretation, the spatial distribution in these two areas points to areas where hominins exploited beavers.

We plotted the spatial distribution of the 2496 beaver specimens (bones and teeth) within the excavated area (**Supplementary Figure 1**). Interestingly, almost all cut marked specimens hail from either the terrace edge/*Uferplatte* or the area of transition to the alluvial fan/*Schwemmfächer*. In general, the spatial distribution of the beaver remains follows the distribution of larger mammals analysed by Brasser [4].

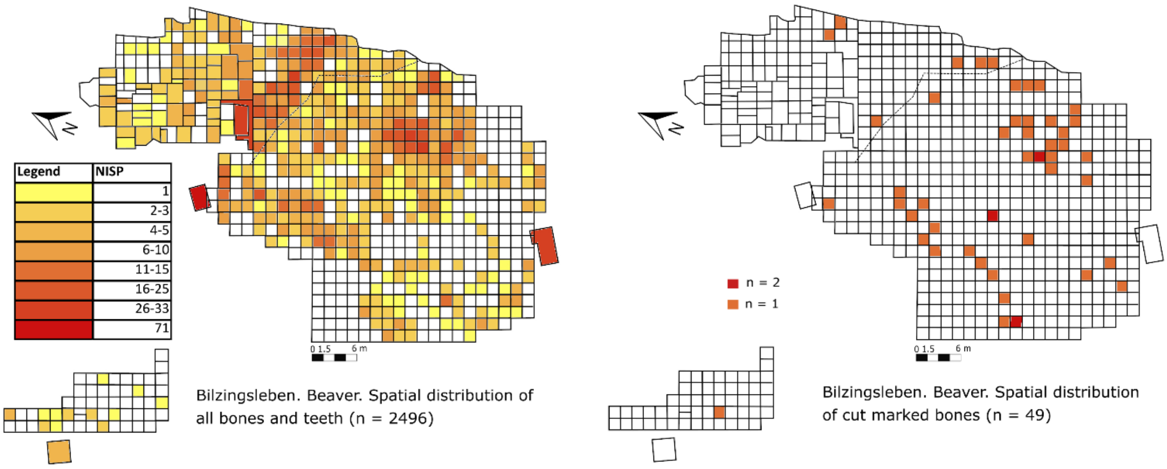

Supplementary Figure 1. Spatial distribution of beaver remains within the 1,5m by 1,5m excavation squares. Left: all beaver remains, Right: distribution of cut marked bones. The thin dashed line indicates the boundary between the alluvial fan (*Schwemmfächer*) in the northern part of the excavated area and the terrace edge (*Uferplatte*) in the south. (See Supplementary Text 1 for further information).

SUPPLEMENTARY TEXT 2: BEAVER EXPLOITATION IN THE EUROPEAN LOWER AND MIDDLE PALAEOLITHIC

There exists some scattered evidence for exploitation of beaver in the later phases of the Middle Palaeolithic, from a small number of sites in Germany, France, Spain, Italy and Croatia. Most data come from a relatively short period, the Last Interglacial, when the temperate climate and forested conditions in major parts of Eurasia provided ideal habitats for beavers. The Eemian "spear site" Lehringen (Germany) yielded one cut marked beaver pelvis fragment [12]. Bratlund [13], studying the faunal material from the Last Interglacial travertine site Taubach (Germany), identified 10 cut marked beaver bone fragments from an assemblage with an MNI of 17 [13], twice the amount of cut marked bones recovered from contemporaneous deposits in the Grand Abri aux Puces in France [14]. The Last Interglacial Layer 1 at Krapina (Croatia) (beaver MNI 19, NISP 353) also yielded some cut marked *Castor fiber* remains [15]. At other Middle Palaeolithic sites evidence consists of very small numbers in MNI for beaver as well as for cut marked bones. Examples come from Pech de l'Azé IV, France with one cut marked bone[16], two cut marked bones at Pech de l'Azé I (level 7) [17], and rare occurrences of cut marked beaver bones in two final Middle Palaeolithic sites in northeast Italy[18]. For the Lower Palaeolithic, data are virtually absent: some beaver remains from Gruta Aroeira and Gruta da Oliveira (Portugal), from archaeological deposits found in association with remains of hunted game and stone tools, may have entered the site through human activities, but there are no cut marks to testify to human interference [19]. Anthropogenic modifications of Lower Palaeolithic beaver remains are in fact limited to in total seven cut marked bones from two levels in the Caune de l'Arago (France), thought to attest to occasional procurement and exploitation of *Castor* around 350-400,000 years ago [20].

		NISP			MNE			MNI			
	specimen	dex	indet	sin	dex	indet	sin		NISPcut	MNEcut	MNIcut
Head	Cranium/Maxilla	1	1	1	1	1	1	2			
	Mandibula	28	9	20	18	1	26	26	6	6	5
	I sup/inf		407								
	I inf	7		9							
	P/M sup	51	64	4	51	64	4				
	P/M sup/inf	1	1143	1	1	1143	1	77-94			
	P/M inf	77	56	143	77	56	143				
Torso, Tail, Zono-skeleton	Vert cerv		1			1		1			
	Vert tho/lumb		3			3		1	1	1	1
	Vert lumb		5			5		2	1	1	1
	Vert lumb/caud		1			1					
	Vert caud		9			9		1			
	Costa	1	17		1	16		1			
	Scapula	7	1	6	6		4	6	1	1	1
	Pelvis	6	3	11	5	2	10	10	2	2	1
Stylo-, Zeugo-podium	Humerus	14	3	20	8	1	12	13	6	5	5
	Radius	7		3	4		3	5	1	1	1
	Ulna	9	2	11	6	2	9	11	4	3	3
	Femur	32	11	27	20		23	23	13	13	8
	Tibia	45	13	50	27	7	33	36	7	6	3
	Fibula		1			1		1			
Auto-podium	Carpals		4			4		1			
	Carpals/Tarsals		1			1		1			
	Astragalus	17		11	17		11	17			
	Calcaneus	12		7	12		6	12	4	4	4
	other Tarsals	3	2	2	3	2	2	2			
	Metapodial	16	19	7	15	8	6	5	3	3	1
	Phalange 1		9			9		1			
	Phalange 2		5			5		1			
	Phalange 3		5			5		1			
other	indet		34								
	**total**		**2496**			**1913**		**77-94**	**49**	**46**	**8**

Supplementary Table 1. Skeletal element representation for the Bilzingsleben beaver material. NISP: Number of Identified Specimens, MNE: Minimum Number of Elements, MNI: Minimum Number of Individuals, NISPcut: Number of Identified Specimens with cut marks, MNEcut: Minimum Number of Elements with cut marks, MNIcut: Minimum Number of Individuals with cut marks, dex: skeletal elements from the right side of the body, sin: skeletal elements from the left side of the body, indet: body side indeterminate.

REFERENCES

1 Mania, D. in *Bilzingsleben - Homo erectus, seine Kultur und seine Umwelt* Vol. I *Veröffentlichungen des Landesamtes für Archäologie in Halle* (eds D. Mania, V. Toepfer, & E. Vlcek) 83-89 (VEB Deutscher Verslag der Wissenschaften, 1980).
2 Mania, D. & Altermann, M. Zur Geologie des altpaläolithischen Fundhorizontes von Bilzingsleben (Thüringen) unter Berücksichtigung des geologischen Wirkfaktors "Mensch". *Hercynia N.F.* **37**, 143-184 (2004).
3 Müller, W. & Pasda, C. Site formation and faunal remains of the Middle Pleistocene site Bilzingsleben. *Quartär* **58**, 25-49 (2011).
4 Brasser, M. *Die Megafauna von Bilzingsleben*. (Landesamt für Denkmalpflege und Archäologie, 2017).
5 Vlcek, E., Mania, D. & Mania, U. Der fossile Mensch von Bilzingsleben. *Bilzingsleben* **VI**, 1-392 (2002).
6 Hublin, J. J. The origin of Neandertals. *Proc. Natl Acad. Sci. USA* **106**, 16022-16027 (2009).
7 Daniel, T. & Frenzel, P. Pleistocene freshwater ostracods from the *Homo erectus* site at Bilzingsleben, Germany—Review of historic collection and unpublished manuscript material for palaeoenvironmental reconstruction. *Geoarchaeology* **38**, 445-465, (2023).
8 Heinrich, W.-D. & Maul, L. C. Mortality profiles of *Castor* and *Trogontherium* (Mammalia: Rodentia, Castoridae), with notes on the site formation of the Mid-Pleistocene hominin locality Bilzingsleben II (Thuringia, Central Germany). *Fossil Imprint* **76**, 40-58 (2020).
9 Rabinovich, R., Gaudzinski-Windheuser, S., Kindler, L. & Goren-Inbar, N. *The Acheulian Site of Gesher Benot Ya'aqov Volume III: Mammal Taphonomy—The Assemblages of Layers V-5 and V-6*. (Springer Netherlands, Dordrecht, 2012).
10 Gaudzinski-Windheuser, S., Kindler, L., Rabinovich, R. & Goren-Inbar, N. Testing heterogeneity in faunal assemblages from archaeological sites. Tumbling and trampling experiments at the early-Middle Pleistocene site of Gesher Benot Ya'aqov (Israel). *Journal of Archaeological Science* **37**, 3170-3190 (2010).
11 Heinrich, W. D. in *Bilzingsleben V. Beiträge zur Ur- und Frühgeschichte Mitteleuropas 4* Vol. 121-134 (ed D. Mania) (Beier & Beran, 2004).
12 Gaudzinski, S. A Matter of High Resolution? The Eemian Interglacial (OIS 5e) in North-central Europe and Middle Palaeolithic Subsistence. *International Journal of Osteoarchaeology* **14**, 201-211 (2004).
13 Bratlund, B. Taubach Revisited. *Jahrbuch des Römisch-Germanischen Zentralmuseums Mainz* **46**, 61-174 (1999).
14 Slimak, L. *et al.* Le Grand Abri aux Puces, a Mousterian site from the Last Interglacial: paleogeography, paleoenvironment, and new excavation results. *Journal of Archaeological Science* **37**, 2747-2761 (2010).
15 Miracle, P. T. *The Krapina Paleolithic site: Zooarchaeology, taphonomy, and catalog of the faunal remains*. (Croatian Natural History Museum, 2007).
16 Dibble, H. L. *et al.* A Preliminary Report on Pech de l'Azé IV, Layer 8 (Middle Paleolithic, France). *PaleoAnthropology* **2009**, 182-219 (2009).
17 Rendu, W. Hunting behavior and Neanderthal adaptability in the Late Pleistocene site of Pech-de-l'Azé I. *Journal of Archaeological Science* **37**, 1798-1810, (2010).

18 Romandini, M. *et al.* Late Neandertals and the exploitation of small mammals in northern Italy: fortuity, necessity or hunting variability? *Quaternaire. Revue de l'Association française pour l'étude du Quaternaire* **29**, 61-67 (2018).

19 Cuenca-Bescós, G., Sanz, M., Daura, J. & Zilhão, J. The fossils of *Castor fiber* from the middle Pleistocene site of Gruta da Aroeira (Portugal) and human-beaver interaction. *Quaternaire. Revue de l'Association française pour l'étude du Quaternaire* **32**, 01-10 (2021).

20 Lebreton, L., Moigne, A.-M., Filoux, A. & Perrenoud, C. A specific small game exploitation for Lower Paleolithic: The beaver (*Castor fiber*) exploitation at the Caune de l'Arago (Pyrénées-Orientales, France). *Journal of Archaeological Science: Reports* **11**, 53-58, (2017).
